# Supplementary material for: Structural basis for oligoclonal T cell recognition of a shared p53 cancer neoantigen
Source: Nat Commun. 2020 Jun 9;11:2908. doi: 10.1038/s41467-020-16755-y (PMC7283474; doi:10.1038/s41467-020-16755-y)
Supplement: Supplementary file 1 — Supplementary Information [file 41467_2020_16755_MOESM1_ESM.pdf]

Supplementary Information

Structural basis for oligoclonal T cell response to a shared p53 cancer neoantigen

Daichao Wu et al.

Supplementary Tables 1–9

Supplementary Figures 1–3

Supplementary Table 1. Data collection and structure refinement statistics

	**p53R175H–HLA-A2**	**p53–HLA-A2**
PDB accession code	6VR5	6VR1
**Data collection**		
Resolution range (Å)[a]	48.1–2.38 (2.47–2.38)	39.8–2.37 (2.46–2.37)
Space group	$P12_11$	$P12_11$
Unit cell parameters	71.6 Å, 79.7 Å, 85.9 Å<br>90°, 102.1°, 90°	71.3 Å, 79.7 Å, 85.8 Å<br>90°, 101.7°, 90°
Total reflections[a]	186,546 (18,604)	184,901 (17,529)
Unique reflections[a]	37,930 (3,742)	38,306 (3,796)
Multiplicity[a]	4.9 (5.0)	4.8 (4.6)
Completeness (%)[a]	99.8 (99.9)	99.6 (99.5)
Mean $I/\sigma(I)$[a]	13.7 (2.9)	12.0 (2.2)
Wilson B factor (Å^2)	32.6	37.4
R_{merge}[a,b]	0.108 (0.415)	0.109 (0.566)
$CC_{1/2}$[a]	0.994 (0.900)	0.995 (0.849)
**Refinement**		
Resolution range (Å)	20.0–2.38	20.0–2.37
Reflections used in refinement[a]	37,921 (3,743)	38,288 (3,797)
R_{work}[a,c]	0.229 (0.290)	0.217 (0.290)
R_{free}[a,c]	0.280 (0.328)	0.274 (0.332)
No. of protein atoms	6,149	6,164
No. of waters	243	350
Protein residues	765	768
r.m.s.d. from ideality		
Bond lengths (Å)	0.012	0.013
Bond angles (°)	1.94	2.07
Ramachandran plot statistics		
Favored (%)	98.0	96.4
Allowed (%)	1.9	3.3
Disallowed (%)	0.1	0.3
Rotamer outliers (%)	2.4	3.7
Clashscore	7.3	9.4
Average B factor (Å^2)	33.0	41.0
Protein	33.0	40.8
Waters	35.3	48.5

[a]Values in parentheses correspond to the highest resolution shell.

[b]$R_{merge} = \sum|I_j - \langle I \rangle|/\sum I_j$, where I_j is the intensity of an individual reflection and $\langle I \rangle$ is the average intensity of that reflection.

[c]R_{work} (R_{free}) = $\sum||F_o| - |F_c||/\sum|F_o|$; 5.0% of data were used for R_{free}.

Supplementary Table 2. Data collection and refinement statistics

	TCR 12-6–p53R175H–HLA-A2	TCR 38-10–p53R175H–HLA-A2	TCR 1a2–p53R175H–HLA-A2
PDB accession code	6VRM	6VRN	6QVO
**Data collection**			
Resolution range (Å)	37.9–2.61 (2.70–2.61)	42.9–2.46 (2.55–2.46)	48.5–3.00 (3.11–3.00)
Space group	*C*121	*P*212121	*P*31
Unit cell parameters	89.4 Å, 120.6 Å, 119.7 Å<br>90°, 108.4°, 90°	44.2 Å, 87.4 Å, 245.9 Å<br>90°, 90°, 90°	118.1 Å, 118.1 Å, 153.1 Å<br>90°, 90°, 120°
Total reflections[a]	94,817 (9,927)	179,362 (17,356)	122,552 (12,329)
Unique reflections[a]	36,118 (3,613)	34,706 (3,222)	47,558 (4,418)
Multiplicity[a]	2.6 (2.7)	5.2 (5.2)	2.6 (2.6)
Completeness (%)[a]	98.1 (99.0)	96.6 (93.7)	97.2 (92.8)
Mean $I/\sigma(I)$[a]	12.1 (1.9)	7.8 (2.5)	7.4 (2.3)
Wilson *B* factor (Å^2)	64.5	36.5	56.4
R_{merge}[a,b]	0.044 (0.343)	0.145 (0.604)	0.110 (0.477)
$CC_{1/2}$ [a]	0.998 (0.945)	0.989 (0.806)	0.844 (0.538)
**Refinement**			[d]see below
Resolution range (Å)	19.8–2.61	20.0–2.46	48.5–3.00
Reflections used in refinement[a]	36,009 (3,613)	34,529 (3,222)	47,254 (4,421)
R_{work}[c]	0.191 (0.389)	0.194 (0.270)	0.162 (0.327)
R_{free}[c]	0.275 (0.407)	0.259 (0.343)	0.211 (0.366)
No. of protein atoms	6,282	6,465	12,740
No. of waters	21	294	
Protein residues	809	813	1,614
r.m.s.d. from ideality			
Bond lengths (Å)	0.011	0.011	0.008
Bond angles (°)	1.88	1.77	1.71
Ramachandran plot statistics			
Favored (%)	90.7	96.1	90.8
Allowed (%)	8.5	3.6	8.2
Disallowed (%)	0.8	0.3	1.0
Rotamer outliers (%)	8.2	4.5	9.3
Clashscore	13.0	5.8	13.6
Average *B* factor (Å^2)	78.0	39.0	54.0
Protein	78.0	40.0	54.0
Waters	81.5	38.9	

[a]Values in parentheses correspond to the highest resolution shell.

[b]$R_{merge} = \sum|I_j - \langle I \rangle|/\sum I_j$, where I_j is the intensity of an individual reflection and $\langle I \rangle$ is the average intensity of that reflection.

[c]R_{work} (R_{free}) $= \sum||F_o| - |F_c||/\sum|F_o|$; 5.0% of data were used for R_{free}.

[d]This structure was twinned and refined with two domains, where the minor domain, with twin law (K, H, L), has twin factor 0.39.

Supplementary Table 3. Data collection and refinement statistics

	**TCR 12-6**	**TCR 1a2**
PDB accession code	6VTH	6VTC
**Data collection**		
Resolution range (Å)[a]	42.5–2.36 (2.44–2.36)	35.4–1.83 (1.90–1.83)
Space group	*P*1211	*P*22121
Unit cell parameters	65.6 Å, 85.0 Å, 88.7 Å<br>90°, 93.6°, 90°	41.1 Å, 106.4 Å, 112.7 Å<br>90°, 90°, 90°
Total reflections[a]	99,994 (10,557)	340,502 (33,949)
Unique reflections[a]	36,570 (3,696)	44,449 (4,388)
Multiplicity	2.7 (2.8)	7.7 (7.7)
Completeness (%)[a]	91.1 (92.6)	99.7 (99.8)
Mean $I/\sigma(I)$	13.8 (3.9)	30.6 (4.7)
Wilson *B* factor (Å^2)	35.2	34.6
R_{merge}[a,b]	0.096 (0.263)	0.081 (0.328)
$CC_{1/2}$[a]	0.978 (0.828)	0.996 (0.973)
**Refinement**		
Resolution range (Å)	20.0–2.36	20.0–1.83
Reflections used in refinement[a]	36,570 (3,696)	44,437 (4,388)
R_{work}[a,c]	0.212 (0.208)	0.190 (0.265)
R_{free}[a,c]	0.264 (0.256)	0.231 (0.314)
No. of protein atoms	6,694	3,433
No. of waters	347	391
Protein residues	867	440
r.m.s.d. from ideality		
Bond lengths (Å)	0.008	0.015
Bond angles (°)	1.51	2.23
Ramachandran plot statistics		
Favored (%)	97.5	96.8
Allowed (%)	2.5	2.8
Disallowed (%)	0.0	0.4
Rotamer outliers (%)	2.8	3.71
Clashscore	9.4	11.1
Average *B* factor (Å^2)	40.0	43.0
Protein	40.0	42.2
Waters	44.9	52.7

[a]Values in parentheses correspond to the highest resolution shell.

[b]$R_{merge} = \sum|I_j - \langle I\rangle|/\sum I_j$, where I_j is the intensity of an individual reflection and $\langle I\rangle$ is the average intensity of that reflection.

[c]R_{work} (R_{free}) $= \sum||F_o| - |F_c||/\sum|F_o|$; 5.0% of data were used for R_{free}.

Supplementary Table 4. TCR center positions over peptide-MHC plane for MHC class I complexes

Complex[1]	*x* pos[2]	*y* pos[2]
5SWS	22.3	-14.8
5SWZ	22.3	-13.9
38-10	14.8	-6.1
3TJH	13.8	-3.3
5TEZ	13	-0.8
3TF7	12.9	3
3PQY	12.5	1.6
4G9F	12.5	-4.8
3TFK	12.4	2.5
4G8G	12.2	-4.6
3KPR	11.8	-1.4
3KPS	11.8	-1.3
4N0C	11.3	1.6
4N5E	11.3	1.8
5IVX	10.9	1.6
1MI5	10.6	-2.8
2OL3	10.6	0.1
5W1W	10.4	-0.5
4MXQ	10.3	2.9
1NAM	10	-1.8
5W1V	10	-0.6
12-6	10.0	-2.3
1FO0	9.6	-0.4
5D2L	8.8	-1.7
3TPU	8.1	0.1
1a2	8.1	-0.3
4MS8	8	1.8
3GSN	7.6	-3.2
2OI9	7.4	2.2
5M01	7.4	-2.2
6G9Q	7.4	-2.2
5M00	7.2	-1.8
5D2N	7.1	-2.5
5HHO	7.1	4.9
3E3Q	7	2
5EUO	7	4.6
3E2H	6.9	1.8
2E7L	6.8	1.9

4MVB	6.8	1.2
5M02	6.8	-2.1
5TIL	6.6	-2.1
2VLR	6.5	4.5
5HHM	6.5	4.7
5E6I	6.4	6.5
5TJE	6.4	-2.2
2ESV	6.3	2.4
3SJV	6.2	-2.5
1G6R	6	1.2
1MWA	5.9	1.5
2BNQ	5.9	-1.9
1OGA	5.8	4
2PYE	5.8	-3.6
4EUP	5.8	1.3
5EU6	5.8	1.1
6MTM	5.8	1
2BNR	5.7	-2.1
2P5E	5.7	-3.5
2P5W	5.6	-3.5
5YXU	5.6	-2.7
2YPL	5.5	1.2
4MJI	5.4	1.9
5WLG	5.3	1.5
5JZI	5.2	-1.7
2F53	5	-3
3QDM	4.9	-2.3
5E9D	4.8	5.3
5ISZ	4.6	3.9
5JHD	4.5	4.9
6BJ2	4.4	5.6
5MEN	4.1	5.1
1LP9	4	2.7
4NHU	4	-5.7
4MNQ	3.9	5.2
3DXA	3.6	-1.5
3RGV	3.4	-1.2
4PRH	3.4	3
3MV7	3.1	2.4
3MV8	3.1	2.6

3MV9	3.1	2.7
4PRP	3	2.6
3VXM	2.7	-3.4
5NHT	2.7	1.1
5NQK	2.4	0.9
5NMG	2.2	3.2
3VXS	2.1	-0.6
5C0B	2.1	-1.9
3QEQ	2	-0.8
6AVG	2	1.2
3O4L	1.7	-1.2
5C0C	1.7	-1.7
5NME	1.7	3.1
6D78	1.7	-0.2
4L3E	1.6	-0.4
3VXR	1.5	-0.6
5C08	1.4	-2.8
5C0A	1.4	-0.8
3QDG	1.3	-1
5C09	1.3	-1.5
5WKF	1.3	-1.3
6DKP	1.3	-0.2
2AK4	1.2	6.6
3QDJ	1.2	-0.9
5C07	1.2	-1.7
5NMF	1.2	3.2
5WKH	1.2	-1.6
3VXU	1.1	3.3
5HYJ	1	-2.3
6AM5	0.9	-0.8
4JRX	0.8	8.5
1KJ2	0.7	2.5
2GJ6	0.6	1.8
3UTS	0.4	-0.2
6EQA	0.2	-1.5
4JFD	0.1	-2.5
5BRZ	0.1	6.4
5BS0	0.1	6.8
3QFJ	0	1.7
6EQB	0	-2.2

3PWP	-0.1	1.6
4JFE	-0.1	-2.4
4JFF	-0.1	-2.4
4QOK	-0.1	-1.8
6AMU	-0.1	0.2
1QRN	-0.2	2
3H9S	-0.2	2
3HG1	-0.2	-1.9
4FTV	-0.3	1.6
1AO7	-0.4	2
1QSE	-0.5	1.4
2NX5	-0.7	2.4
1QSF	-0.8	1.5
4QRP	-0.9	-1.3
1BD2	-1.3	-0.3
6AVF	-1.7	-0.1
3FFC	-2.3	2.9
4JRY	-14.6	0.8

[1]PDB code for complex structure, with new structures described in this work given by TCR name (12-6, 38-10, 1a2) and corresponding rows highlighted.

[2]TCR–pMHC complexes were oriented into a common reference frame centered at average Cα atom position of MHC helices, and rotated such that the *x*-*y* plane is parallel with the helix plane, and the *x*-axis is parallel to peptide groove, with greater *x* value corresponding to peptide C-terminus. TCR variable domain centers were calculated by taking centers of individual variable domains by average positions of Sγ atoms of conserved Cys residues (or Cα atoms at corresponding positions where Cys residues are not present in the TCR), and then calculating the mean position of TCR Vα and Vβ centers. *X* position (*x* pos) and *y* position (*y* pos) values represent projections into the *x*-*y* plane, and thus the MHC helix plane, of these centers.

Supplementary Table 5. Interactions between TCRs and HLA-A2

HLA-A2	TCR 12-6		TCR 38-10		TCR 1a2	
	Hydrogen bonds	Van der Waals contacts	Hydrogen bonds	Van der Waals contacts	Hydrogen bonds	Van der Waals contacts
α1						
R65H	G94α(O) R65H(Nη2), Q96α(NE2) R65H(Nε)	G94α(7), G93α(1), Y95α(1), Q96α(9)		S28α(2)	L94α(O) R65H(Nη2)	A29α(3), L94α(3), E96α(2)
K68H		Y95α(2)			E96α(O) K68H(O)	E96α(4)
A69H		Y95α(5), W98β(4)		S98α(1)		L94α(3), K95α(1), S98α(1)
Q72H		Y95α(3), W98β(9)				E96α(1), D97α(5), S98α(2)
T73H		W98β(2)				S98α(1)
R75H	N30β(Oδ1) R75H(Nη2), S51β(Oγ) R75H(Nη1)	N30β(2), S51β(2)		D56β(1)		
V76H		N30β(1)		Y50β(1)		
T80H				R30β(2)		
α2						
K146H			R30β(Nη2) K146H(Nζ)	R30β(2), L96β(2)		
A149H				L96β(1)		D100β(1)
A150H		Y51α(5)	Y97α(OH) A150H(O)	Y97α(5)		
H151H		Y51α(18)	E52α(Oε2) H151H(Nδ1), K55α(Nζ) H151H(Nε2)	E52α(5), Y54α(1), K55α(5)		Y51α(2)
V152H				Y97α(3)		
E154H	S52α(N) E154(Oε1), S53α(Oγ) E154(Oε1), S53α(Oγ) E154(Oε2), S53α(N) E154(Oε1)	Y51α(4), S52α(3), S53α(6)		Y54α(3)		Y51α(4)
Q155H	S32α(Oγ) Q155H(Oε1), Q31α(Nε2) Q155H(Oε1)	Q31α(3), Y51α(1), V100β(2)	N31α(Nδ2) Q155H(Oε1), N31α(Nδ2) Q155H(Nε2), Y97α(OH) Q155H(Nε2)	N31α(3), Y54α(2), Y97α(3)	Q31α(Oε1) Q155H(Nε2), Y32α(OH) Q155H(Oε1), Y32α(OH) Q155H(Nε2)	Y32α(7), Y51α(6)

Contact residues were identified with CONTACT (43). Hydrogen bonds were calculated using a cut-off distance of 3.5 Å. The cut-off distance for van der Waals contacts was 4.0 Å.

Supplementary Table 6. Interactions between TCRs and p53R175H peptide

p53R175H	TCR 12-6		TCR 38-10		TCR 1a2	
	Hydrogen bonds	Van der Waals contacts	Hydrogen bonds	Van der Waals contacts	Hydrogen bonds	Van der Waals contacts
E4p	G94α(N) E4p(Oε1)	V100β(1), G93α(2)	S98α(Oγ) E4p(O), N30α(N) E4p(Oε2)	E29α(3), N30α(8), S98α(3)	Y100α(OH) E4p(O)	A29α(6), Q31α(1), L94α(2), Y100α(3)
V5p				Y97α(1)		Y100α(1)
V6p	V100β(N) V6p(O)	W98β(1), Q99β(3), V100β(1)		S98α(1)	Q97β(Nε2) V6p(O)	L94α(1), Y100α(2)
R7p	Q99β(Oε1) R7p(Nε) V100β(O) R7p(Nη2), G101β(O) R7p(Nη2) E103β(Oε1) R7p(Nη1)	Q99β(12), V100β(3), G101β(2), E103β(5)	Y97α(O) R7p(Nη2), L96β(O) R7p(Nη1), V97β(O) R7p(Nη1)	Y97α(10), Y103α(12), V97β(1), T98β(1)	D100β(Oδ2) R7p(Nη1), Y32α(OH) R7p(Nη1), D100β(Oδ2) R7p(Nη2)	Q96β(2), Q97β(4), A99β(3), D100β(2), Y32α(3), Y100α(1)
H8p	E95β(Oε2) H8p(Nδ1), W98β(Nε1) H8p(Nε2), Q99β(Oε1) H8p(N)	E95β(5), G96β(2), W98β(3), Q99β(10)	Y103α(OH) H8p(N), Y31β(OH) H8p(Nδ1)	Y31β(5), R30β(3), Y50β(1), Y103α(24)	Q97β(Oε1) H8p(N), Q96β(Nε2) H8p(O), S98α(Oγ) H8p(Nε2)	M50β(1), Q96β(4), Q97β(15), S98α(2)
C9p			R30β(Nη2) C9p(O)	R30β(1)		

Contact residues were identified with CONTACT (43). Hydrogen bonds were calculated using a cut-off distance of 3.5 Å. The cut-off distance for van der Waals contacts was 4.0 Å.

Supplementary Table 7. Calculated ΔΔ*G* of peptide point mutations based on TCR complex structures

Peptide mutant	12-6[1]	38-10[1]	1a2[1]
H1A	0.0	0.0	0.0
M2A	0.0	0.0	0.0
T3A	0.2	0.2	0.2
E4A	1.3	1.5	1.2
V5A	0.7	0.4	0.3
V6A	0.4	0.2	0.3
R7A	2.3	3.6	2.2
H8A	1.7	2.8	2.4
C9A	0.0	0.0	0.0
H8R	1.6	2.0	1.2

[1]Calculated binding affinity change for interaction with pMHC for the indicated TCR based on analysis of corresponding TCR–pMHC complex structures in Rosetta, as described in Methods. Values are given in Rosetta Energy Units, which correspond approximately to energy in kcal/mol.

Supplementary Table 8. Rosetta scoring term contributions to calculated TCR binding energy change (ΔΔ*G*) for H8R reversion.

Term name	Description	12-6	38-10	1a2
fa_atr	attractive van der Waals	**2.1**	-0.3	-0.5
fa_rep	repulsive van der Waals	-0.1	0.8	0.0
fa_sol	desolvation	-0.5	0.3	0.3
hbond_lr_bb	backbone hydrogen bond	0.0	0.0	0.0
hbond_bb_sc	backbone-side chain hydrogen bond	0.0	0.0	0.0
hbond_sc	side chain-side chain hydrogen bond	0.0	**1.2**	**1.4**
Total ΔΔ*G*		1.6	2.0	1.2

Values are in Rosetta Energy Units (REU), and values from dominant term are shown in bold for each interface. All terms are weighted according to the "interface" scoring function in Rosetta.

Supplementary Table 9. Sequences of codon-optimized TCR genes

Name	Sequence
12-6 alpha	ATGCGTAAAGAAGTTGAGCAGGATCCGGGTCCGTTCAACGTGCCGGAGGGTGCGACCGTT<br>GCGTTCAACTGCACCTACAGCAACAGCGCGAGCCAGAGCTTCTTTTGGTACCGTCAAGAT<br>TGCCGTAAGGAGCCGAAACTGTTGATGAGCGTTTACTCCAGCGGTAACGAAGACGGCCGT<br>TTCACCGCGCAGCTGAACCGTGCGAGCCAATACATCAGCTTGCTGATTCGCGACAGCAAA<br>CTGAGCGATAGCGCGACCTACCTGTGCGTGGTTCAGCCGGGTGGCTACCAAAAGGTGACC<br>TTCGGTACCGGTACCAAACTGCAGGTTATCCCGAACATTCAGAACCCGGACCCGGCGGTG<br>TACCAACTGCGTGACAGCAAGAGCTCCGATAAAAGCGTGTGCCTGTTCACCGACTTCGAT<br>AGCCAGACCAACGTTAGCCAAAGCAAGGACAGCGATGTGTACATCACCGACAAATGCGTT<br>CTGGATATGCGTAGCATGGACTTCAAGAGCAATAGCGCTGTGGCGTGGAGCAACAAGAGC<br>GATTTCGCGTGCGCGAACGCGTTCAACAATAGCATCATTCCGGAGGACACCTTCTTTCCG<br>AGCCCGGAAAGCTCCTAA
12-6 beta	ATGAACGCGGGCGTGACCCAGACCCCGAAGTTCCAAGTTCTGAAAACCGGTCAGAGCATG<br>ACCCTGCAGTGCGCGCAAGACATGAACCACAACAGCATGTACTGGTATCGTCAAGATCCG<br>GGTATGGGCCTGCGCCTGATCTACTATAGCGCGAGCGAGGGTACCACTGACAAGGGCGAA<br>GTGCCGAACGGTTACAACGTTAGCCGTCTGAACAAGCGTGAGTTCAGCCTGCGTCTGGAA<br>AGCGCGGCTCCGAGCCAGACCAGCGTGTACTTCTGCGCGAGCTCCGAGGGCCTGTGGCAG<br>GTTGGTGACGAACAATACTTCGGTCCGGGTACCCGTCTGACCGTGACCGAGGATCTGAAG<br>AACGTTTTCCCACCGGAAGTGGCGGTTTTCGAACCGAGCGAGGCGGAAATTAGCCACACC<br>CAGAAAGCGACCCTGGTGTGCCTGGCGACCGGCTTCTATCCGGACCACGTGGAGCTGTCC<br>TGGTGGGTTAACGGCAAGGAAGTGCACAGCGGTGTTTGCACCGACCCGCAGCCGCTGAAA<br>GAGCAACCGGCGCTGAACGATAGCCGTTATGCGCTGAGCTCCCGTCTGCGTGTGAGCGCG<br>ACCTTCTGGCAGAACCCGCGTAACCACTTCCGTTGCCAGGTTCAATTCTATGGCCTGAGC<br>GAGAACGACGAATGGACCCAGGATCGTGCGAAGCCGGTGACCCAAATCGTTAGCGCGGAA<br>GCGTGGGGTCGTGCGGATTAA
38-10 alpha	ATGGCGCAGACCGTTACCCAAAGCCAACCGGAGATGAGCGTGCAAGAGGCGGAAACCGTT<br>ACCCTGAGCTGCACCTACGATACCAGCGAAAACAATTATTACCTGTTCTGGTACAAGCAG<br>CCACCGAGCCGTCAAATGATCCTGGTGATTCGTCAGGAAGCGTACAAACAGCAAAACGCG<br>ACCGAAAACCGTTTCAGCGTGAACTTCCAGAAGGCCGCGAAGAGCTTCAGCCTGAAGATC<br>AGCGACAGCCAACTGGGTGATACCGCGATGTATTTCTGCGCGTTCATGGGCTACAGCGGT<br>GCGGGCAGCTATCAGCTGACCTTTGGCAAGGGCACCAAACTGAGCGTGATCCCGAACATT<br>CAGAACCCGGACCCGGCGGTTTACCAACTGCGTGACAGCAAGAGCTCCGATAAAAGCGTG<br>TGCCTGTTCACCGACTTCGATAGCCAGACCAACGTTAGCCAAAGCAAGGACAGCGATGTG<br>TACATCACCGACAAGTGCGTTCTGGATATGCGTAGCATGGACTTCAAGAGCAACAGCGCG<br>GTTGCGTGGAGCAATAAAAGCGATTTCGCGTGCGCGAACGCGTTCAACAATAGCATCATT<br>CCGGAGGACACCTTCTTTCCGAGCCCGGAAAGCTCCTAA

38-10 beta	ATGGATGCCGGTATTACCCAAAGCCCGCGTCACAAGGTGACCGAAACCGGTACCCCGGTT<br>ACCCTGCGTTGCCACCAGACCGAAAACCACCGTTACATGTACTGGTACCGTCAAGATCCG<br>GGTCACGGCCTGCGTCTGATTCACTATAGCTACGGTGTGAAGGACACCGACAAAGGTGAG<br>GTGAGCGACGGCTATAGCGTTAGCCGTAGCAAAACCGAGGATTTCCTGTTGACCCTGGAA<br>AGCGCGACCAGCTCCCAGACCAGCGTGTACTTCTGCGCGATCAGCGAGCTGGTTACCGGT<br>GACAGCCCGCTGCACTTTGGTAACGGCACCCGTCTGACCGTGACCGAAGATCTGAAGAAC<br>GTTTTCCCACCGGAAGTGGCGGTTTTCGAACCGAGCGAGGCGGAAATTAGCCACACCCAG<br>AAAGCGACCCTGGTTTGCCTGGCGACCGGTTTCTATCCGGACCACGTGGAGCTGTCCTGG<br>TGGGTTAACGGCAAGGAAGTGCATAGCGGCGTTTGCACCGACCCGCAGCCGCTGAAAGAG<br>CAACCGGCGCTGAACGATAGCCGTTATGCGCTGAGCTCCCGTCTGCGTGTGAGCGCGACC<br>TTCTGGCAAAACCCGCGTAACCACTTCCGTTGCCAGGTTCAATTCTACGGTCTGAGCGAG<br>AACGACGAATGGACCCAGGATCGTGCGAAGCCGGTGACCCAAATTGTTAGCGCGGAAGCG<br>TGGGGTCGTGCCGACTAA
1a2 alpha	ATGCAGAAAGAAGTGGAACAGGATCCGGGTCCGCTGAGCGTGCCGGAAGGTGCGATTGTG<br>AGCCTGAACTGCACCTACAGCAACAGCGCGTTTCAGTATTTCATGTGGTATCGTCAGTAT<br>AGCCGTAAAGGTCCGGAACTGCTCATGTATACCTATAGCAGCGGCAACAAGGAAGATGGC<br>CGTTTCACCGCTCAAGTGGATAAGAGCAGCAAATACATTAGCCTGTTCATTCGTGACAGC<br>CAGCCGAGCGATAGCGCGACCTATCTGTGCGCGATGAGCGGCCTGAAGGAGGACAGCAGC<br>TATAAACTGATCTTTGGCAGCGGTACCCGTCTGCTGGTTCGTCCGGATATTCAAAACCCG<br>GACCCGGCGGTGTACCAACTGCGTGATAGCAAGAGCAGCGATAAAAGCGTGTGCCTGTTC<br>ACCGACTTCGATAGCCAGACCAACGTTAGCCAAAGCAAGGACAGCGATGTGTATATCACC<br>GACAAATGCGTTCTGGATATGCGTAGCATGGACTTCAAGAGCAATAGCGCGGTTGCGTGG<br>AGCAACAAAAGCGATTTCGCGTGCGCGAACGCGTTTAACAACAGCATCATTCCGGAGGAC<br>ACCTTCTTTCCGAGCCCGGAAAGCAGCTAA
1a2 beta	ATGGAAGCGCAGGTGACCCAGAACCCGCGCTATCTGATTACCGTGACCGGCAAAAAACTG<br>ACCGTGACCTGCAGCCAGAACATGAACCATGAATATATGAGCTGGTATCGCCAGGATCCG<br>GGCCTGGGCCTGCGCCAGATTTATTATAGCATGAACGTGGAAGTGACCGATAAAGGCGAT<br>GTGCCGGAAGGCTATAAAGTGAGCCGCAAAGAAAAACGCAACTTTCCGCTGATTCTGGAA<br>AGCCCGAGCCCGAACCAGACCAGCCTGTATTTTTGCGCGAGCAGCATTCAGCAGGGCGCG<br>GATACCCAGTATTTTGGCCCGGGCACCCGCCTGACCGTGCTGGAAGATCTGAAGAACGTT<br>TTCCCACCGGAAGTGGCGGTTTTCGAACCGAGCGAGGCGGAAATTAGCCACACCCAGAAA<br>GCGACCCTGGTTTGCCTGGCGACCGGTTTCTATCCGGACCACGTGGAGCTGTCCTGGTGG<br>GTTAACGGCAAGGAAGTGCATAGCGGCGTTTGCACCGACCCGCAGCCGCTGAAAGAGCAA<br>CCGGCGCTGAACGATAGCCGTTATGCGCTGAGCTCCCGTCTGCGTGTGAGCGCGACCTTC<br>TGGCAAAACCCGCGTAACCACTTCCGTTGCCAGGTTCAATTCTACGGTCTGAGCGAGAAC<br>GACGAATGGACCCAGGATCGTGCGAAGCCGGTGACCCAAATTGTTAGCGCGGAAGCGTGG<br>GGTCGTGCCGACTAA

Supplementary Fig. 1

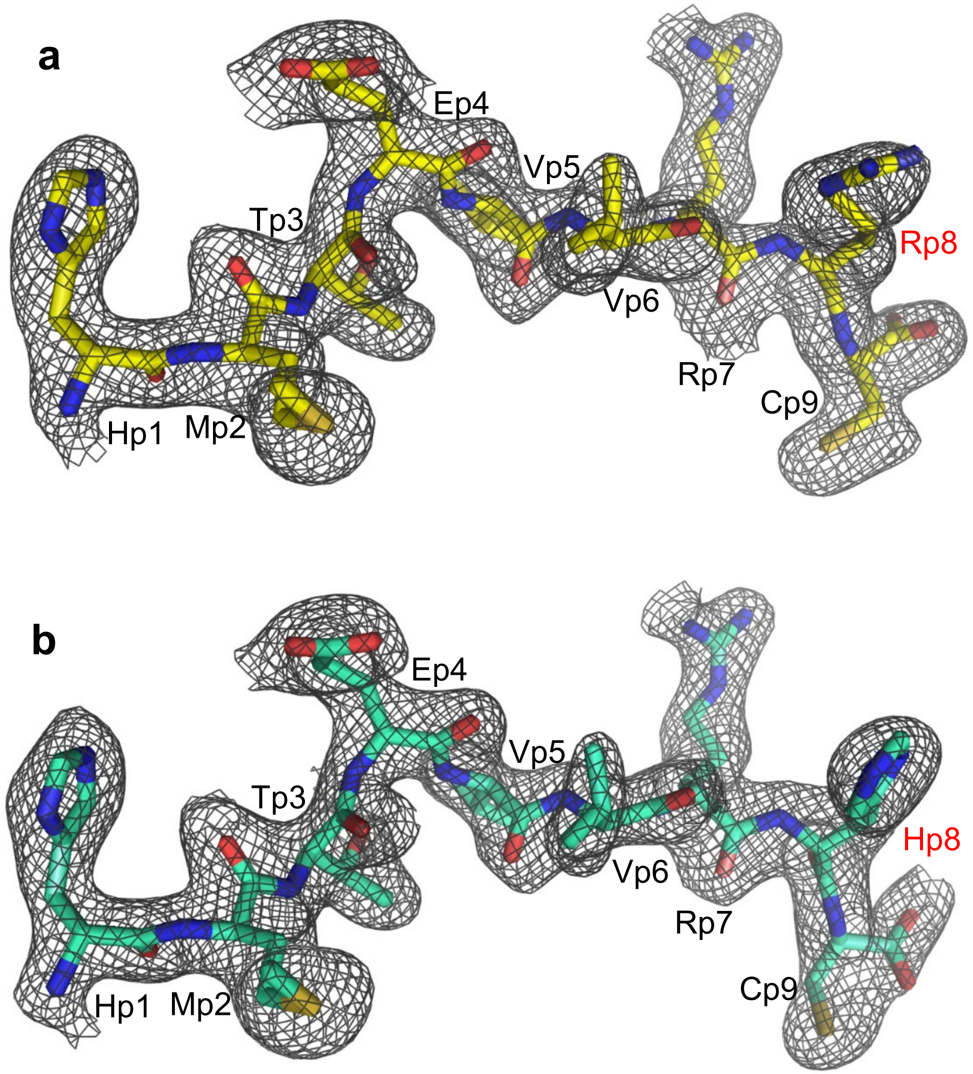

Supplementary Figure 1. (a) Electron density for the bound wild-type p53 peptide in the p53–HLA-A2 complex. The $F_o - F_c$ omit map at 2.37 Å resolution is contoured at 1σ. **(b)** Electron density for the bound mutant p53R175H peptide in the p53R175H–HLA-A2 complex. The $F_o - F_c$ omit map at 2.38 Å resolution is contoured at 1σ.

Supplementary Fig. 2

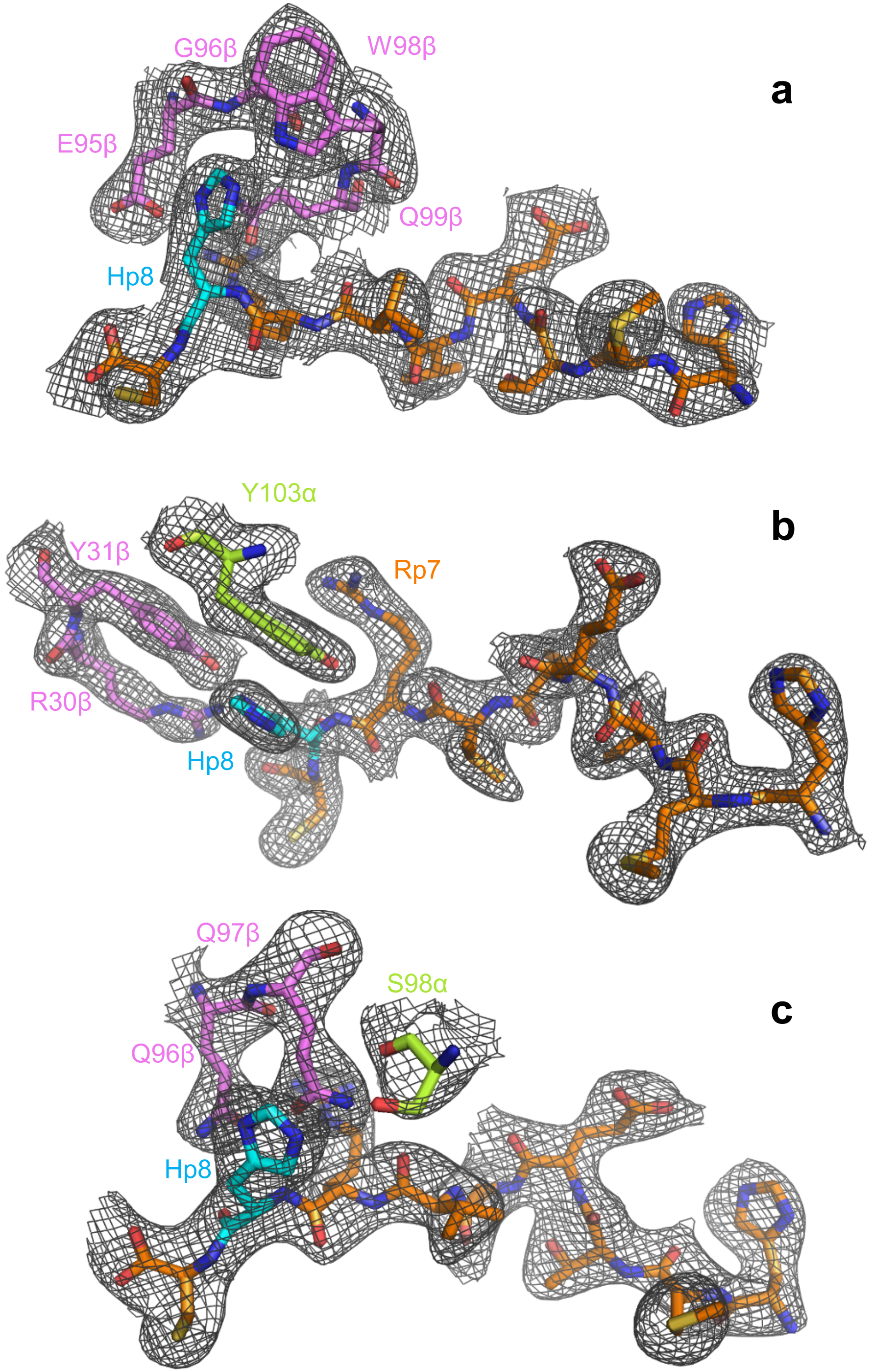

Supplementary Figure 2. (a) Electron density at the interface in the 12-6–p53R175H–HLA-A2 complex. Density from the final $2F_o - F_c$ map at 2.61 Å resolution is contoured at 1σ. **(b)** Electron density at the interface in the 38-10–p53R175H–HLA-A2 complex. Density from the final $2F_o - F_c$ map at 2.46 Å resolution is contoured at 1σ. **(c)** Electron density at the interface in the 1a2–p53R175H–HLA-A2 complex. Density from the final $2F_o - F_c$ map at 3.00 Å resolution is contoured at 1σ.

Supplementary Fig. 3

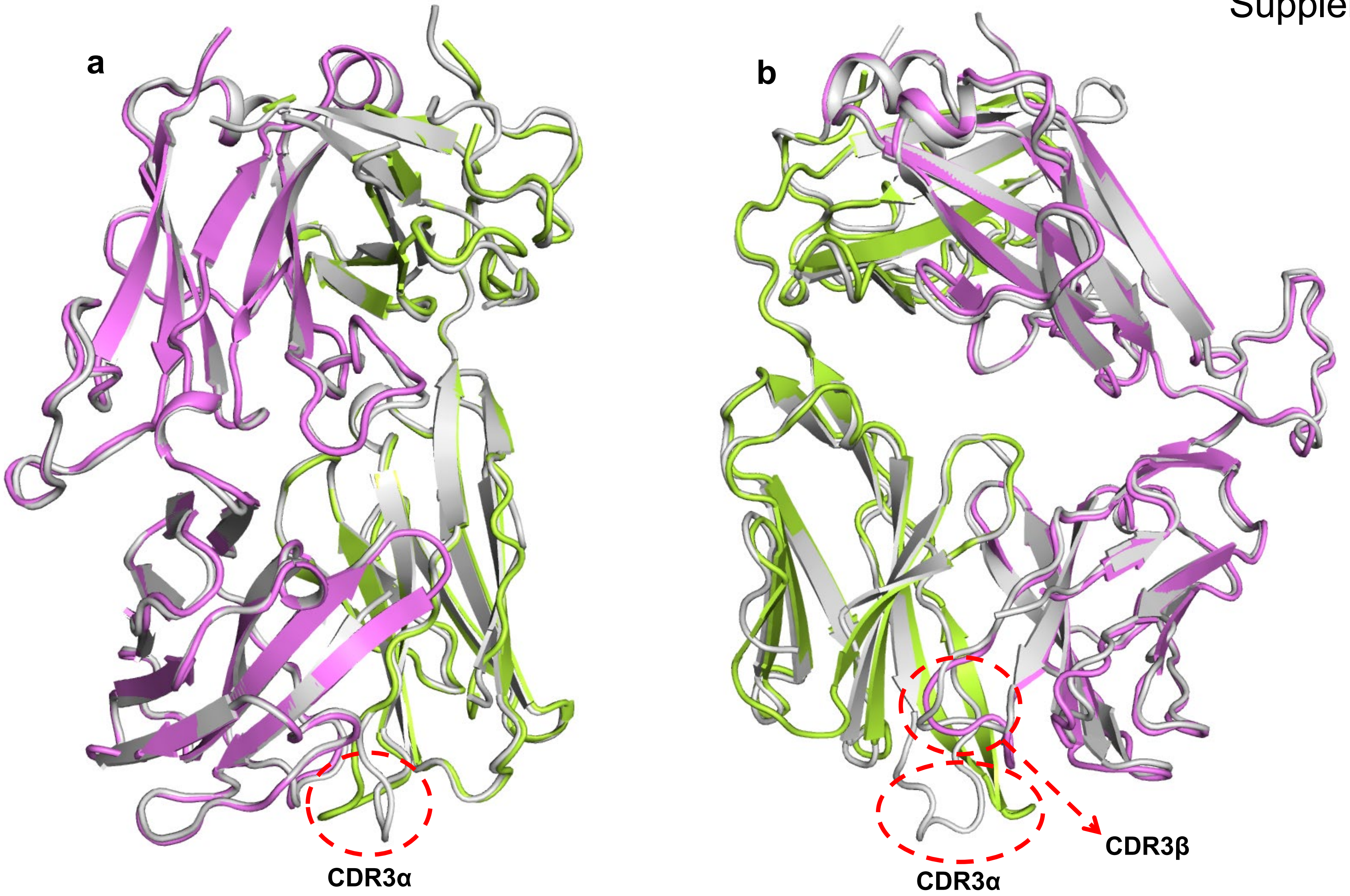

Supplementary Figure 3. (a) Superposition of free TCR 12-6 (gray) onto bound TCR 12-6 (α chain, green; β chain, violet).
(b) Superposition of free TCR 1a2 onto bound TCR 1a2. CDR loops undergoing the largest conformation changes upon binding p53R175H–HLA-A2 are circled.
